# Crystal structures of 2-amino-2-oxoethyl 4-bromo­benzoate, 2-amino-2-oxoethyl 4-nitro­benzoate and 2-amino-2-oxoethyl 4-amino­benzoate monohydrate

**DOI:** 10.1107/S2056989020014371

**Published:** 2020-11-03

**Authors:** F. A. Sapayev, R. Ya. Okmanov, T. S. Kholikov, Kh. S. Tadjimukhamedov, B. Tashkhodjaev

**Affiliations:** a National University of Uzbekistan named after Mirzo Ulugbek, 100174, Massif Universitet Shakharchasi 4, Tashkent, Uzbekistan; b S. Yunusov Institute of the Chemistry of Plant Substances, Academy of Sciences of Uzbekistan 100170, Mirzo Ulugbek Str., 77, Tashkent, Uzbekistan

**Keywords:** 2-amino-2-oxoethyl 4-bromo­benzoate, 2-amino-2-oxoethyl 4-nitro­benzoate and 2-amino-2-oxoethyl 4-amino­benzoate monohydrate, crystal structure, mol­ecular structure, hydrogen bonding

## Abstract

The title mol­ecules were synthesized by the reaction of 4-substituted sodium benzoates with chloro­acetic acid amide in the presence of di­methyl­formamide. The yields of 2-amino-2-oxoethyl 4-bromo­benzoate, C_9_H_8_BrNO_3_, **I**, 2-amino-2-oxoethyl 4-nitro­benzoate, C_9_H_8_N_2_O_5_, **II**, and 2-amino-2-oxoethyl 4-amino­benzoate monohydrate, C_9_H_10_N_2_O_3_·H_2_O, **III**, are 86, 78 and 88%, respectively. The low yield of **II** is explained by the reduced reactivity of the mol­ecule in a nucleophilic exchange reaction because of the negative induction and negative mesomeric effects of the nitro group on the benzene ring. Single crystals were obtained from the products under the same (temperature and solvent) conditions. In the case of **III**, the crystals formed as a monohydrate. In all three crystal structures, the same type of inter­molecular N—H⋯O hydrogen bonds are observed, but the mol­ecules differ in some torsion angles as well as in the dihedral angles between the mean planes of the benzene rings and the amide groups.

## Abstract 2

The title mol­ecules were synthesized by the reaction of the corresponding sodium benzoate with chloro­acetic acid amide. Single crystals were obtained from the reaction products under the same conditions.

## Chemical context   

Mol­ecules containing an aromatic ring, a carboxyl and an amino group represent an important class of organic compounds and, with several reaction centers, they are important inter­mediates in industry. They are often used as synthons in organic synthesis and are also widely used as ligands in the coordination chemistry of various transition metals. These ligands can form a variety of complex compounds as they possess several Lewis base sites.
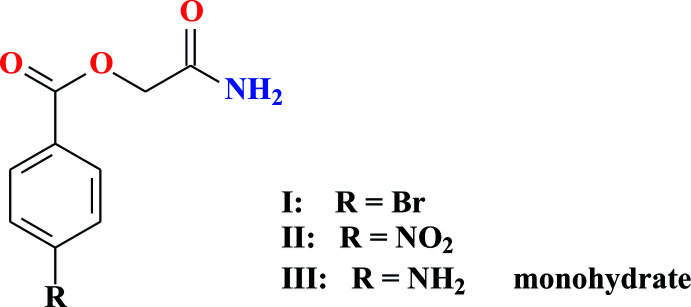


**Figure 1:**
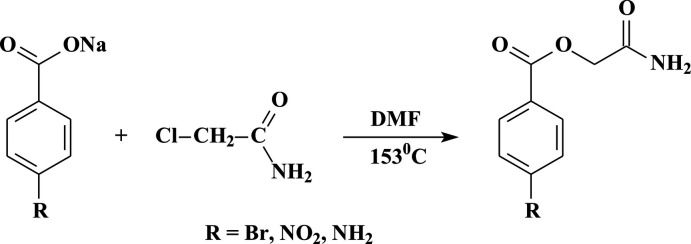
Reaction scheme for the synthesis of (2-amino-2-oxoeth­yl)benzoate derivatives.

The new crystalline compounds 2-amino-2-oxoethyl 4-bromo­benzoate (**I**), 2-amino-2-oxoethyl 4-nitro­benzoate (**II**) and 2-amino-2-oxoeth­yl)-4-amino­benzoate monohydrate (**III**) (Fig. 1 ▸) were synthesized from the reaction of 4-substituted sodium benzoates with chloro­acetic acid amide in the presence of di­methyl­formamide. Their structures were determined by X-ray crystallographic analysis.

## Structural commentary   

**Figure 2:**
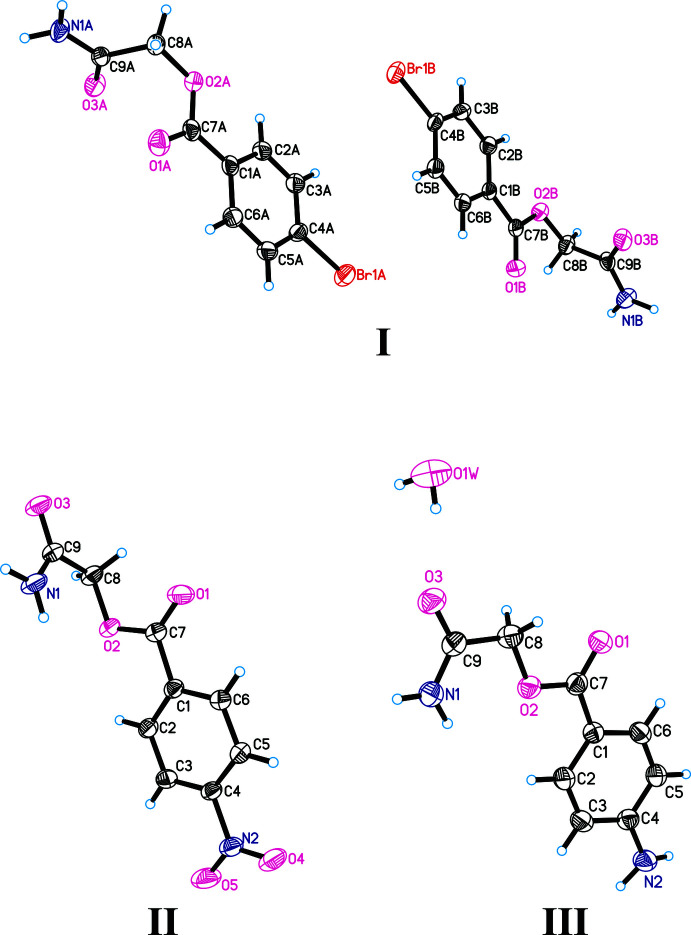
The asymmetric units of **I**–**III** with atom labelling. Displacement ellipsoids are drawn at the 30% probability level.

All of the title structures have planar benzoate (C1–C7/O1/O2) and amide (O3/C9/N1) units but the dihedral angle between these planes is different in each case because of the torsion angle about the bridging methyl­ene group (C8; Tables 1 ▸–3 ▸ ▸). The asymmetric unit of each crystal structure is illus­trated in Fig. 2 ▸. That of **I** consists of two independent mol­ecules (*A* and *B*), which differ in the position of the amide groups relative to the benzoate (r.m.s. deviations of 0.021 Å for *A* and 0.031 Å for *B*) fragments, as indicated by the dihedral angles of 82.5 (4) and 75.9 (3)° in *A* and *B*, respectively. The asymmetric unit of **II** contains only one mol­ecule of 2-amino-2-oxoethyl 4-nitro­benzoate. The dihedral angle between the mean planes of the amide and the benzoate (r.m.s. deviation = 0.070 Å) groups is 89.4 (2)°. The asymmetric unit of **III** contains one water mol­ecule and one 2-amino-2-oxoethyl 4-amino­benzoate mol­ecule (Fig. 2 ▸). The dihedral angle between the mean planes of the amide and benzoate (r.m.s. deviation = 0.027 Å) groups is 4.4 (5)°. Analysis of the bond lengths and bond angles of **I**–**III** shows slight differences, but these data are in the expected ranges (Allen *et al.*, 1987 ▸).

## Supra­molecular features   

In the crystal structures, several types of inter­molecular inter­actions are observed but all contain inter­molecular N—H⋯O hydrogen bonds.

**Figure 3:**
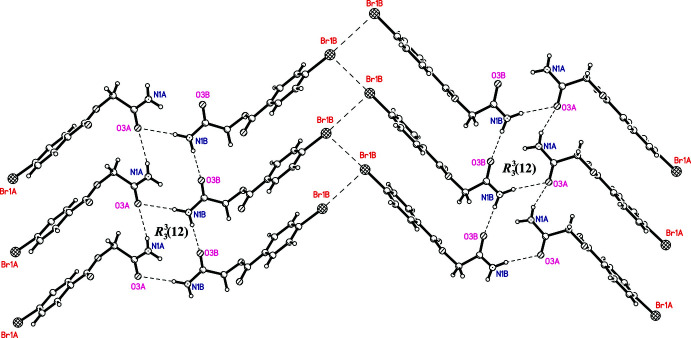
Hydrogen bonds (formation of rings) and inter­molecular Br⋯Br contacts in **I**.

In **I**, inter­molecular *A*⋯*A* (N1*A*—H1*A*⋯O3*A* ^i^), *B*⋯*B* (N1*B*—H2*B*⋯O3*B* ^ii^) and *B*⋯*A* (N1*B*—H2*B*⋯O3*B* ^iii^) inter­actions cross-link the mol­ecules, generating rings with an 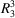
(12) graph-set motif (Fig. 3 ▸, Table 4 ▸) (Grell *et al.*, 1999 ▸). Although both the *A* and *B* mol­ecules contain a bromine atom, a short inter­molecular Br⋯Br inter­action only occurs between *B* mol­ecules [Br1*B*⋯Br1*B*(−*x* + 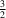, *y* + 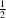, −*z* + 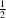) = 3.519 (1) Å, 0.18 Å less than the sum of the van der Waals radii]. This inter­action connects the mol­ecules into chains extending along the *b-*axis direction (Fig. 3 ▸). A similar short Br ⋯ Br inter­action was observed in the crystal structures of 4-chloro­phenyl-4-bromo­benzoate (TAYNEP; Saha & Desiraju, 2017 ▸) and 4-bromo­phenyl-4-bromo­benzoate (VEWSIC; Saha & Desiraju, 2018 ▸).

**Figure 4:**
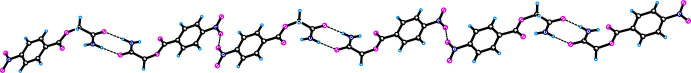
Hydrogen bonds and inter­molecular O⋯O contacts in **II**.

In **II**, the angle between the mean planes of the nitro group and the aromatic ring is 4.1 (1)°. A characteristic inter­molecular inter­action for **II** is the formation of centrosymmetric dimers as a result of the N1—H1⋯O3^i^ hydrogen bonds formed between amide fragments (Table 5 ▸). Short inter­molecular O5⋯O5(−*x* + 1, −*y* + 2, −*z* + 1) inter­actions [at 2.874 (4) Å these are 0.14 Å less than the sum of the van der Waals radii] are observed between the nitro groups of the dimers (Fig. 4 ▸). A similar inter­molecular O⋯O contact was observed in the crystal structure of *meta*-di­nitro­benzene (DNBENZ11, DNBENZ12; Wójcik *et al.*, 2002 ▸).

**Figure 5:**
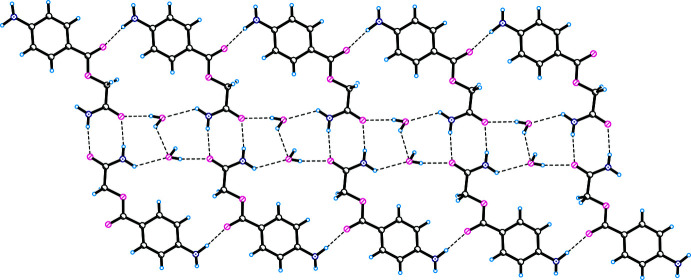
Dimer formation in **III**.

**Figure 6:**
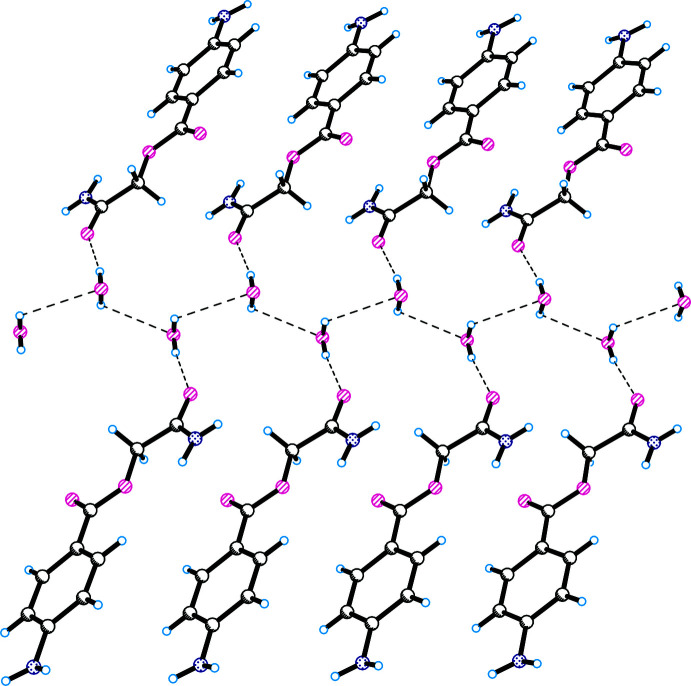
Inter­molecular hydrogen bonds between water and 2-amino-2-oxoethyl 4-amino­benzoate mol­ecules in **III**.

In **III**, as in **II**, inversion dimers are formed by N1—H1⋯O3^i^ hydrogen bonds (Fig. 5 ▸, Table 6 ▸). An inter­molecular hydrogen bond is also observed between the oxygen of the amide fragment and the water mol­ecule (Fig. 6 ▸), although the angle is only 101°, and the water mol­ecules are further connected by hydrogen bonds to form an infinite chain along the *b*-axis direction.

## Database survey   

A search for the 2-amino-2-oxoethyl benzoate (carbamoyl­methyl­benzoate) scaffold in the Cambridge Structural Database (CSD Version 5.41, update of November 2019; Groom *et al.*, 2016 ▸) gave 34 hits. Of these, the structures most closely related to the title compounds are MAMJOC [2-(di­methyl­amino)-2-oxo­ethyl 5-bromo-2-hy­droxy­benzoate; Santra *et al.*, 2016 ▸], CEPWID (1-benzo­yloxy-1-meth­oxy-*N*-methyl­acetamide; Nishio *et al.*, 1984 ▸) and HUMJII (carbamoylmethyl 3,4,5-tri­hydroxy­benzoate hydrate; Parkin *et al.*, 2002 ▸).

## Synthesis and crystallization   

**Synthesis 2-amino-2-oxoethyl 4-bromo­benzoate: General method.** To a 25 mL round-bottom flask containing 0.27 g (1.2 mmol) of the sodium salt of *p*-bromo­benzoic acid were added 6 mL of DMF. The resulting mixture was heated for 10 min (for partial dissolution of the salt) and 0.1 g (1 mmol) of chloro­acetamide was added. The flask was equipped with a reflux condenser and mechanical stirrer and was heated in a sand bath with stirring at 426 K for 6 h. The DMF was removed *in vacuo* (15 mm Hg) at 328 K. After cooling, cold water was poured into the reaction mixture to completely eliminate the residual reactants and DMF. The resulting precipitate was filtered off to give 0.22 g (86% yield) of product. *R*_F_ = 0.65 [in 5:1.5:1 (*v*/*v*) CHCl_3_/C_6_H_6_/CH_3_OH solvent system]; m.p. 475–477 K. ^1^H NMR [400 MHz, CD_3_OD, δ (ppm.), *J* (Hz)]: 7.95 (2H, *d*, *J* = 8.64, H3 and H5), 7.61 (2H, *d*, *J* = 8.63, H2 and H6), 4.72 (2H, *s*, CH_2_).

**(2-Amino-2-oxoeth­yl)-4-nitro­benzoate**. The reaction yield is 78%. *R*_F_ = 0.62 [in 5:1.5:1 (*v*/*v*) CHCl_3_/C_6_H_6_/CH_3_OH solvent system]; m.p. 439–441 K. ^1^H NMR [400 MHz, CD_3_OD+CDCl_3_+C_2_D_5_OD δ (ppm), *J* (Hz)]: 8.23 (2H, *d*, *J* = 9.28, H3 and H5), 8.19 (2H, *d*, *J* = 9.31, H2 and H6), 4.73 (2H, *s*, CH_2_).

**(2-Amino-2-oxoeth­yl)-4-amino­benzoate**. The reaction yield is 88%. *R*_F_ = 0.53 [in 5:1.5:1 (*v*/*v*) CHCl_3_/C_6_H_6_/CH_3_OH solvent system]; m.p. 435–438 K. ^1^H NMR [400 MHz, CD_3_OD, δ (ppm), *J* (Hz)]: 7.75 (2H, *d*, *J* = 8.75, H3 and H5), 6.58 (2H, *d*, *J* = 8.75, H2 and H6), 4.62 (2H, *s*, CH_2_).

Each compound was dissolved in ethanol and the solvent allowed to evaporate at room temperature. Colourless crystals suitable for X-ray diffraction analysis were obtained.

The crystal of the 2-amino-2-oxoethyl 4-amino­benzoate monohydrate loses its transparency without chemical change (without becoming amorphous) in the range 344–346 K when the crystals are heated and melts in the range 435–438 K.

The yields of 2-amino-2-oxoethyl 4-bromo­benzoate, C_9_H_8_BrNO_3_, **I**, 2-amino-2-oxoethyl 4-nitro­benzoate, C_9_H_8_N_2_O_5_, **II**, and 2-amino-2-oxoethyl 4-amino­benzoate monohydrate, C_9_H_10_N_2_O_3_·H_2_O, **III**, are 86, 78 and 88%, respectively. The low yield of **II** is explained by the reduced reactivity of the mol­ecule in a nucleophilic exchange reaction because of the negative induction and negative mesomeric effects of the nitro group on the benzene ring.

## Refinement   

Crystal data, data collection and structure refinement details are summarized in Table 7 ▸. C-bound H atoms were placed geometrically (with C—H distances of 0.97 Å for CH_2_ and 0.93 Å for C_ar_) and included in the refinement as riding contributions with *U*_iso_(H) = 1.2*U*_eq_(C) [*U*_iso_ = 1.5 *U*_eq_(C) for methyl H atoms]. The hydrogen atoms attached to N and O (water) were located in difference-Fourier maps and refined freely.

## Supplementary Material

Crystal structure: contains datablock(s) I, II, III, Global. DOI: 10.1107/S2056989020014371/mw2171sup1.cif

Structure factors: contains datablock(s) I. DOI: 10.1107/S2056989020014371/mw2171Isup2.hkl

Structure factors: contains datablock(s) II. DOI: 10.1107/S2056989020014371/mw2171IIsup3.hkl

Structure factors: contains datablock(s) III. DOI: 10.1107/S2056989020014371/mw2171IIIsup4.hkl

Supporting information file. DOI: 10.1107/S2056989020014371/mw2171Isup5.cml

Supporting information file. DOI: 10.1107/S2056989020014371/mw2171IIsup6.cml

Supporting information file. DOI: 10.1107/S2056989020014371/mw2171IIIsup7.cml

CCDC references: 2041177, 2041176, 2041175

Additional supporting information:  crystallographic information; 3D view; checkCIF report

## Figures and Tables

**Table 1:** Selected torsion angles (°) for (I)

C8*A*—O2*A*—C7*A*—C1*A*	179.4 (3)	C8*B*—O2*B*—C7*B*—C1*B*	177.1 (3)
C6*A*—C1*A*—C7*A*—O2*A*	178.3 (3)	C6*B*—C1*B*—C7*B*—O2*B*	−176.7 (3)
C7*A*—O2*A*—C8*A*—C9*A*	−72.9 (5)	C7*B*—O2*B*—C8*B*—C9*B*	−69.1 (5)
O2*A*—C8*A*—C9*A*—O3*A*	−16.9 (6)	O2*B*—C8*B*—C9*B*—O3*B*	−17.6 (6)

**Table 2:** Selected torsion angles (°) for (II)

C8—O2—C7—C1	−176.52 (14)	C7—O2—C8—C9	−95.53 (19)
C6—C1—C7—O2	−170.76 (15)	O2—C8—C9—O3	175.79 (17)

**Table 3:** Selected torsion angles (°) for (III)

C8—O2—C7—C1	−178.9 (2)	C7—O2—C8—C9	−179.2 (2)
C6—C1—C7—O2	−177.0 (2)	O2—C8—C9—O3	177.4 (2)

**Table 4:** Hydrogen-bond geometry (Å, °) for (I)

*D*—H⋯*A*	*D*—H	H⋯*A*	*D*⋯*A*	*D*—H⋯*A*
N1*A*—H1*A*⋯O3*A* ^i^	0.85 (6)	2.18 (7)	2.969 (5)	153
N1*B*—H1*B*⋯O3*A* ^ii^	0.93 (6)	2.24 (6)	3.163 (5)	170
N1*B*—H2*B*⋯O3*B* ^iii^	0.86 (9)	1.97 (9)	2.825 (5)	178

Symmetry codes: (i) 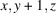; (ii) 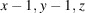; (iii) 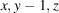.

**Table 5:** Hydrogen-bond geometry (Å, °) for (II)

*D*—H⋯*A*	*D*—H	H⋯*A*	*D*⋯*A*	*D*—H⋯*A*
N1—H1⋯O3^i^	0.93 (2)	1.97 (2)	2.898 (2)	174 (2)

Symmetry code: (i) 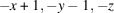.

**Table 6:** Hydrogen-bond geometry (Å, °) for (III)

*D*—H⋯*A*	*D*—H	H⋯*A*	*D*⋯*A*	*D*—H⋯*A*
N1—H1⋯O3^i^	0.87 (4)	2.06 (4)	2.915 (3)	168
N2—H3⋯O1^ii^	0.96 (3)	1.98 (3)	2.919 (4)	163
O1*W*—H1*W*⋯O3	0.78 (4)	2.14 (4)	2.916 (4)	169 (4)
O1*W*—H2*W*⋯O1*W* ^iii^	0.91 (9)	2.46 (9)	2.782 (7)	101

Symmetry codes: (i) 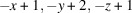; (ii) 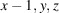; (iii) 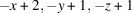.

**Table 7:** Experimental details

	(I)	(II)	(III)
Crystal data			
Chemical formula	C_9_H_8_BrNO_3_	C_9_H_8_N_2_O_5_	C_9_H_10_N_2_O_3_·H_2_O
*M*_r_	258.07	224.17	212.21
Crystal system, space group	Monoclinic, *P*2_1_/*n*	Triclinic, *P* 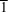	Monoclinic, *P*2_1_/*n*
Temperature (K)	291	291	291
*a*, *b*, *c* (Å)	18.623 (4), 4.8255 (10), 23.195 (5)	7.1238 (14), 7.3683 (15), 10.063 (2)	8.2431 (16), 4.8088 (10), 26.754 (5)
α, β, γ (°)	90, 112.96 (3), 90	107.82 (3), 94.95 (3), 96.32 (3)	90, 90.10 (3), 90
*V* (Å^3^)	1919.3 (8)	495.76 (19)	1060.5 (4)
*Z*	8	2	4
Radiation type	Cu *K*α	Cu *K*α	Cu *K*α
μ (mm^−1^)	5.71	1.08	0.90
Crystal size (mm)	0.60 × 0.20 × 0.15	0.40 × 0.34 × 0.21	0.28 × 0.24 × 0.17

Data collection			
Diffractometer	Oxford Diffraction Xcalibur, Ruby	Oxford Diffraction Xcalibur, Ruby	Oxford Diffraction Xcalibur, Ruby
Absorption correction	Multi-scan (*SADABS*; Bruker, 2008 ▸)	Multi-scan (*SADABS*; Bruker, 2008 ▸)	Multi-scan (*SADABS*; Bruker, 2008 ▸)
*T*_min_, *T*_max_	0.292, 0.425	0.681, 0.797	0.778, 0.859
No. of measured, independent and observed [*I* > 2σ(*I*)] reflections	6390, 3832, 3165	2971, 1859, 1560	6444, 2165, 1129
*R*_int_	0.033	0.018	0.055
(sin θ/λ)_max_ (Å^−1^)	0.629	0.609	0.630

Refinement			
*R*[*F* ^2^ > 2σ(*F* ^2^)], *wR*(*F* ^2^), *S*	0.049, 0.138, 1.06	0.050, 0.148, 1.06	0.053, 0.150, 0.99
No. of reflections	3832	1859	2165
No. of parameters	269	153	160
H-atom treatment	H atoms treated by a mixture of independent and constrained refinement	H atoms treated by a mixture of independent and constrained refinement	H atoms treated by a mixture of independent and constrained refinement
Δρ_max_, Δρ_min_ (e Å^−3^)	0.62, −0.85	0.24, −0.29	0.16, −0.24

Computer programs: *CrysAlis PRO* (Rigaku OD, 2018 ▸), *SHELXS7* (Sheldrick, 2008 ▸), *SHELXL2014/8* (Sheldrick, 2015 ▸), *XP* in *SHELXTL* (Sheldrick, 2008 ▸), *SHELXTL* (Sheldrick, 2008 ▸), *PLATON* (Spek, 2020 ▸) and *publCIF* (Westrip, 2010 ▸)’.

## supplementary crystallographic information

### 2-Amino-2-oxoethyl 4-bromobenzoate (I) Crystal data

**Table:**

C_9_H_8_BrNO_3_	*D*_x_ = 1.786 Mg m^−^^3^
*M**_r_* = 258.07	Melting point: 475(2) K
Monoclinic, *P*2_1_/*n*	Cu *K*α radiation, λ = 1.54184 Å
*a* = 18.623 (4) Å	Cell parameters from 2381 reflections
*b* = 4.8255 (10) Å	θ = 3.9–75.4°
*c* = 23.195 (5) Å	µ = 5.71 mm^−^^1^
β = 112.96 (3)°	*T* = 291 K
*V* = 1919.3 (8) Å^3^	Prismatic, colorless
*Z* = 8	0.60 × 0.20 × 0.15 mm
*F*(000) = 1024	

### 2-Amino-2-oxoethyl 4-bromobenzoate (I) Data collection

**Table:**

Oxford Diffraction Xcalibur, Ruby diffractometer	3832 independent reflections
Radiation source: Enhance (Cu) X-ray Source	3165 reflections with *I* > 2σ(*I*)
Graphite monochromator	*R*_int_ = 0.033
Detector resolution: 10.2576 pixels mm^-1^	θ_max_ = 75.9°, θ_min_ = 3.9°
ω scans	*h* = −20→22
Absorption correction: multi-scan (SADABS; Bruker, 2008)	*k* = −3→5
*T*_min_ = 0.292, *T*_max_ = 0.425	*l* = −28→28
6390 measured reflections	

### 2-Amino-2-oxoethyl 4-bromobenzoate (I) Refinement

**Table:**

Refinement on *F*^2^	Primary atom site location: structure-invariant direct methods
Least-squares matrix: full	Secondary atom site location: difference Fourier map
*R*[*F*^2^ > 2σ(*F*^2^)] = 0.049	Hydrogen site location: mixed
*wR*(*F*^2^) = 0.138	H atoms treated by a mixture of independent and constrained refinement
*S* = 1.06	*w* = 1/[σ^2^(*F*_o_^2^) + (0.0772*P*)^2^ + 0.3454*P*] where *P* = (*F*_o_^2^ + 2*F*_c_^2^)/3
3832 reflections	(Δ/σ)_max_ = 0.001
269 parameters	Δρ_max_ = 0.62 e Å^−^^3^
0 restraints	Δρ_min_ = −0.85 e Å^−^^3^

### 2-Amino-2-oxoethyl 4-bromobenzoate (I) Special details

**Table:**

Geometry. All esds (except the esd in the dihedral angle between two l.s. planes) are estimated using the full covariance matrix. The cell esds are taken into account individually in the estimation of esds in distances, angles and torsion angles; correlations between esds in cell parameters are only used when they are defined by crystal symmetry. An approximate (isotropic) treatment of cell esds is used for estimating esds involving l.s. planes.

### 2-Amino-2-oxoethyl 4-bromobenzoate (I) Fractional atomic coordinates and isotropic or equivalent isotropic displacement parameters (Å^2^)

**Table:**

	*x*	*y*	*z*	*U*_iso_*/*U*_eq_	
Br1A	0.59795 (3)	0.16726 (12)	0.02621 (3)	0.06387 (18)	
O1A	0.8638 (2)	1.0616 (8)	−0.01058 (15)	0.0605 (8)	
O2A	0.89019 (17)	1.1199 (6)	0.09136 (15)	0.0503 (7)	
O3A	1.03189 (18)	0.9293 (6)	0.09906 (17)	0.0565 (8)	
N1A	1.0666 (2)	1.3499 (8)	0.0755 (2)	0.0562 (10)	
C1A	0.7921 (2)	0.7970 (9)	0.03619 (17)	0.0415 (8)	
C2A	0.7811 (2)	0.7232 (10)	0.09046 (18)	0.0480 (9)	
H2AA	0.8124	0.8027	0.1285	0.058*	
C3A	0.7248 (2)	0.5349 (10)	0.08845 (19)	0.0490 (9)	
H3AA	0.7173	0.4880	0.1246	0.059*	
C4A	0.6792 (2)	0.4160 (9)	0.03104 (19)	0.0448 (8)	
C5A	0.6903 (3)	0.4759 (9)	−0.0231 (2)	0.0502 (10)	
H5AA	0.6607	0.3889	−0.0606	0.060*	
C6A	0.7466 (2)	0.6684 (10)	−0.02010 (18)	0.0490 (9)	
H6AA	0.7544	0.7128	−0.0563	0.059*	
C7A	0.8509 (2)	1.0019 (9)	0.03547 (19)	0.0446 (9)	
C8A	0.9478 (2)	1.3163 (9)	0.0930 (2)	0.0513 (10)	
H8AA	0.9637	1.4220	0.1316	0.062*	
H8AB	0.9257	1.4444	0.0583	0.062*	
C9A	1.0189 (2)	1.1759 (8)	0.08903 (19)	0.0423 (8)	
Br1B	0.67568 (2)	0.97787 (9)	0.22125 (2)	0.05283 (17)	
O1B	0.38919 (19)	−0.0158 (7)	0.13654 (16)	0.0566 (8)	
O2B	0.40704 (17)	0.0563 (7)	0.23657 (14)	0.0499 (7)	
O3B	0.2552 (2)	0.2216 (6)	0.17521 (19)	0.0599 (8)	
N1B	0.2115 (2)	−0.2140 (8)	0.1638 (2)	0.0590 (10)	
C1B	0.4827 (2)	0.3192 (8)	0.19559 (18)	0.0403 (8)	
C2B	0.5228 (2)	0.4425 (8)	0.25363 (18)	0.0438 (8)	
H2BA	0.5112	0.3927	0.2877	0.053*	
C3B	0.5798 (2)	0.6380 (8)	0.26086 (18)	0.0431 (8)	
H3BA	0.6070	0.7184	0.2998	0.052*	
C4B	0.5961 (2)	0.7133 (8)	0.20969 (19)	0.0413 (8)	
C5B	0.5564 (2)	0.5973 (9)	0.15118 (19)	0.0463 (9)	
H5BA	0.5673	0.6527	0.1170	0.056*	
C6B	0.5005 (2)	0.3988 (9)	0.14437 (18)	0.0451 (8)	
H6BA	0.4743	0.3166	0.1055	0.054*	
C7B	0.4222 (2)	0.1058 (9)	0.18490 (19)	0.0433 (8)	
C8B	0.3457 (2)	−0.1403 (9)	0.2275 (2)	0.0499 (9)	
H8BA	0.3449	−0.1889	0.2679	0.060*	
H8BB	0.3562	−0.3078	0.2090	0.060*	
C9B	0.2666 (2)	−0.0281 (8)	0.1857 (2)	0.0430 (8)	
H1A	1.064 (4)	1.527 (14)	0.074 (3)	0.070 (18)*	
H2A	1.107 (3)	1.286 (12)	0.067 (2)	0.058 (14)*	
H1B	0.160 (4)	−0.159 (12)	0.142 (3)	0.064 (16)*	
H2B	0.225 (5)	−0.385 (19)	0.167 (4)	0.11 (3)*	

### 2-Amino-2-oxoethyl 4-bromobenzoate (I) Atomic displacement parameters (Å^2^)

**Table:**

	*U*^11^	*U*^22^	*U*^33^	*U*^12^	*U*^13^	*U*^23^
Br1A	0.0482 (3)	0.0654 (3)	0.0756 (3)	−0.0109 (2)	0.0215 (2)	−0.0061 (2)
O1A	0.0569 (19)	0.074 (2)	0.0558 (17)	0.0015 (16)	0.0275 (14)	0.0065 (16)
O2A	0.0425 (15)	0.0511 (16)	0.0623 (16)	−0.0055 (12)	0.0258 (13)	−0.0114 (14)
O3A	0.0476 (16)	0.0373 (15)	0.087 (2)	0.0071 (13)	0.0286 (16)	0.0083 (15)
N1A	0.0413 (19)	0.039 (2)	0.096 (3)	0.0026 (15)	0.0345 (19)	0.0011 (19)
C1A	0.0327 (17)	0.048 (2)	0.0439 (17)	0.0086 (15)	0.0146 (14)	−0.0008 (16)
C2A	0.042 (2)	0.057 (2)	0.0425 (18)	0.0013 (18)	0.0139 (15)	−0.0077 (17)
C3A	0.043 (2)	0.059 (3)	0.0448 (19)	0.0018 (18)	0.0166 (16)	−0.0040 (18)
C4A	0.0346 (18)	0.041 (2)	0.054 (2)	−0.0005 (15)	0.0117 (15)	−0.0046 (17)
C5A	0.045 (2)	0.053 (2)	0.048 (2)	0.0029 (18)	0.0125 (17)	−0.0100 (18)
C6A	0.041 (2)	0.061 (3)	0.0414 (18)	0.0027 (18)	0.0131 (15)	−0.0046 (18)
C7A	0.0373 (19)	0.045 (2)	0.053 (2)	0.0099 (16)	0.0200 (16)	0.0010 (17)
C8A	0.039 (2)	0.043 (2)	0.077 (3)	−0.0009 (16)	0.0283 (19)	−0.010 (2)
C9A	0.0335 (17)	0.038 (2)	0.0526 (19)	0.0053 (14)	0.0141 (15)	−0.0037 (16)
Br1B	0.0354 (2)	0.0468 (3)	0.0738 (3)	−0.00022 (16)	0.01852 (19)	0.0072 (2)
O1B	0.0513 (17)	0.0560 (18)	0.0640 (18)	−0.0109 (14)	0.0240 (14)	−0.0166 (15)
O2B	0.0429 (15)	0.0532 (16)	0.0554 (15)	−0.0074 (13)	0.0211 (12)	−0.0041 (13)
O3B	0.0531 (17)	0.0311 (15)	0.097 (2)	0.0056 (13)	0.0305 (17)	0.0047 (15)
N1B	0.048 (2)	0.0341 (19)	0.080 (3)	0.0025 (15)	0.0084 (18)	0.0062 (18)
C1B	0.0310 (16)	0.0385 (19)	0.0511 (19)	0.0056 (14)	0.0155 (14)	−0.0013 (15)
C2B	0.0410 (19)	0.044 (2)	0.0453 (18)	0.0022 (16)	0.0157 (15)	0.0024 (16)
C3B	0.0401 (19)	0.040 (2)	0.0459 (18)	−0.0042 (15)	0.0136 (15)	−0.0016 (15)
C4B	0.0254 (15)	0.0373 (18)	0.056 (2)	0.0002 (13)	0.0106 (14)	0.0024 (16)
C5B	0.0371 (18)	0.054 (2)	0.0483 (19)	0.0046 (17)	0.0166 (15)	0.0018 (17)
C6B	0.0350 (17)	0.052 (2)	0.0453 (18)	0.0046 (16)	0.0124 (14)	−0.0055 (17)
C7B	0.0327 (17)	0.042 (2)	0.054 (2)	0.0067 (15)	0.0152 (15)	−0.0038 (17)
C8B	0.039 (2)	0.044 (2)	0.067 (2)	−0.0004 (16)	0.0206 (18)	0.0046 (19)
C9B	0.043 (2)	0.0319 (19)	0.061 (2)	0.0028 (15)	0.0282 (17)	0.0023 (16)

### 2-Amino-2-oxoethyl 4-bromobenzoate (I) Geometric parameters (Å, º)

**Table:**

Br1A—C4A	1.900 (4)	Br1B—C4B	1.894 (4)
O1A—C7A	1.217 (6)	O1B—C7B	1.201 (5)
O2A—C7A	1.342 (5)	O2B—C7B	1.355 (5)
O2A—C8A	1.421 (5)	O2B—C8B	1.437 (5)
O3A—C9A	1.219 (5)	O3B—C9B	1.231 (5)
N1A—C9A	1.345 (6)	N1B—C9B	1.307 (6)
N1A—H1A	0.85 (7)	N1B—H1B	0.93 (6)
N1A—H2A	0.91 (6)	N1B—H2B	0.86 (9)
C1A—C6A	1.394 (5)	C1B—C2B	1.393 (6)
C1A—C2A	1.398 (6)	C1B—C6B	1.406 (6)
C1A—C7A	1.480 (6)	C1B—C7B	1.474 (6)
C2A—C3A	1.374 (6)	C2B—C3B	1.381 (6)
C2A—H2AA	0.9300	C2B—H2BA	0.9300
C3A—C4A	1.394 (6)	C3B—C4B	1.383 (6)
C3A—H3AA	0.9300	C3B—H3BA	0.9300
C4A—C5A	1.380 (6)	C4B—C5B	1.385 (6)
C5A—C6A	1.382 (7)	C5B—C6B	1.378 (6)
C5A—H5AA	0.9300	C5B—H5BA	0.9300
C6A—H6AA	0.9300	C6B—H6BA	0.9300
C8A—C9A	1.522 (5)	C8B—C9B	1.512 (6)
C8A—H8AA	0.9700	C8B—H8BA	0.9700
C8A—H8AB	0.9700	C8B—H8BB	0.9700
			
C7A—O2A—C8A	115.5 (3)	C7B—O2B—C8B	114.5 (3)
C9A—N1A—H1A	127 (4)	C9B—N1B—H1B	120 (4)
C9A—N1A—H2A	121 (4)	C9B—N1B—H2B	117 (5)
H1A—N1A—H2A	111 (6)	H1B—N1B—H2B	122 (6)
C6A—C1A—C2A	118.7 (4)	C2B—C1B—C6B	119.1 (4)
C6A—C1A—C7A	117.9 (4)	C2B—C1B—C7B	123.2 (4)
C2A—C1A—C7A	123.3 (4)	C6B—C1B—C7B	117.7 (4)
C3A—C2A—C1A	121.1 (4)	C3B—C2B—C1B	120.4 (4)
C3A—C2A—H2AA	119.5	C3B—C2B—H2BA	119.8
C1A—C2A—H2AA	119.5	C1B—C2B—H2BA	119.8
C2A—C3A—C4A	118.5 (4)	C2B—C3B—C4B	119.4 (4)
C2A—C3A—H3AA	120.7	C2B—C3B—H3BA	120.3
C4A—C3A—H3AA	120.7	C4B—C3B—H3BA	120.3
C5A—C4A—C3A	122.1 (4)	C3B—C4B—C5B	121.5 (4)
C5A—C4A—Br1A	118.5 (3)	C3B—C4B—Br1B	118.5 (3)
C3A—C4A—Br1A	119.4 (3)	C5B—C4B—Br1B	120.0 (3)
C4A—C5A—C6A	118.4 (4)	C6B—C5B—C4B	119.0 (4)
C4A—C5A—H5AA	120.8	C6B—C5B—H5BA	120.5
C6A—C5A—H5AA	120.8	C4B—C5B—H5BA	120.5
C5A—C6A—C1A	121.2 (4)	C5B—C6B—C1B	120.7 (4)
C5A—C6A—H6AA	119.4	C5B—C6B—H6BA	119.7
C1A—C6A—H6AA	119.4	C1B—C6B—H6BA	119.7
O1A—C7A—O2A	121.8 (4)	O1B—C7B—O2B	122.2 (4)
O1A—C7A—C1A	124.6 (4)	O1B—C7B—C1B	125.3 (4)
O2A—C7A—C1A	113.6 (4)	O2B—C7B—C1B	112.6 (3)
O2A—C8A—C9A	111.5 (3)	O2B—C8B—C9B	112.1 (3)
O2A—C8A—H8AA	109.3	O2B—C8B—H8BA	109.2
C9A—C8A—H8AA	109.3	C9B—C8B—H8BA	109.2
O2A—C8A—H8AB	109.3	O2B—C8B—H8BB	109.2
C9A—C8A—H8AB	109.3	C9B—C8B—H8BB	109.2
H8AA—C8A—H8AB	108.0	H8BA—C8B—H8BB	107.9
O3A—C9A—N1A	123.7 (4)	O3B—C9B—N1B	123.1 (4)
O3A—C9A—C8A	122.4 (4)	O3B—C9B—C8B	121.8 (4)
N1A—C9A—C8A	113.9 (4)	N1B—C9B—C8B	115.1 (4)
			
C6A—C1A—C2A—C3A	−2.3 (6)	C6B—C1B—C2B—C3B	−0.6 (6)
C7A—C1A—C2A—C3A	178.9 (4)	C7B—C1B—C2B—C3B	178.9 (4)
C1A—C2A—C3A—C4A	0.7 (7)	C1B—C2B—C3B—C4B	0.8 (6)
C2A—C3A—C4A—C5A	1.8 (7)	C2B—C3B—C4B—C5B	0.1 (6)
C2A—C3A—C4A—Br1A	−177.3 (3)	C2B—C3B—C4B—Br1B	−179.1 (3)
C3A—C4A—C5A—C6A	−2.6 (6)	C3B—C4B—C5B—C6B	−1.3 (6)
Br1A—C4A—C5A—C6A	176.5 (3)	Br1B—C4B—C5B—C6B	177.9 (3)
C4A—C5A—C6A—C1A	0.9 (6)	C4B—C5B—C6B—C1B	1.4 (6)
C2A—C1A—C6A—C5A	1.5 (6)	C2B—C1B—C6B—C5B	−0.5 (6)
C7A—C1A—C6A—C5A	−179.7 (4)	C7B—C1B—C6B—C5B	179.9 (4)
C8A—O2A—C7A—O1A	−0.4 (6)	C8B—O2B—C7B—O1B	−3.3 (6)
C8A—O2A—C7A—C1A	179.4 (3)	C8B—O2B—C7B—C1B	177.1 (3)
C6A—C1A—C7A—O1A	−1.9 (6)	C2B—C1B—C7B—O1B	−175.9 (4)
C2A—C1A—C7A—O1A	176.9 (4)	C6B—C1B—C7B—O1B	3.7 (6)
C6A—C1A—C7A—O2A	178.3 (3)	C2B—C1B—C7B—O2B	3.7 (5)
C2A—C1A—C7A—O2A	−3.0 (5)	C6B—C1B—C7B—O2B	−176.7 (3)
C7A—O2A—C8A—C9A	−72.9 (5)	C7B—O2B—C8B—C9B	−69.1 (5)
O2A—C8A—C9A—O3A	−16.9 (6)	O2B—C8B—C9B—O3B	−17.6 (6)
O2A—C8A—C9A—N1A	165.0 (4)	O2B—C8B—C9B—N1B	164.7 (4)

### 2-Amino-2-oxoethyl 4-bromobenzoate (I) Hydrogen-bond geometry (Å, º)

**Table:**

*D*—H···*A*	*D*—H	H···*A*	*D*···*A*	*D*—H···*A*
N1*A*—H1*A*···O3*A*^i^	0.85 (6)	2.18 (7)	2.969 (5)	153
N1*B*—H1*B*···O3*A*^ii^	0.93 (6)	2.24 (6)	3.163 (5)	170
N1*B*—H2*B*···O3*B*^iii^	0.86 (9)	1.97 (9)	2.825 (5)	178

Symmetry codes: (i) *x*, *y*+1, *z*; (ii) *x*−1, *y*−1, *z*; (iii) *x*, *y*−1, *z*.

### 2-Amino-2-oxoethyl 4-nitrobenzoate (II) Crystal data

**Table:**

C_9_H_8_N_2_O_5_	*F*(000) = 232
*M**_r_* = 224.17	*D*_x_ = 1.502 Mg m^−^^3^
Triclinic, *P*1	Melting point: 439(2) K
*a* = 7.1238 (14) Å	Cu *K*α radiation, λ = 1.54184 Å
*b* = 7.3683 (15) Å	Cell parameters from 1249 reflections
*c* = 10.063 (2) Å	θ = 4.6–75.6°
α = 107.82 (3)°	µ = 1.08 mm^−^^1^
β = 94.95 (3)°	*T* = 291 K
γ = 96.32 (3)°	Prismatic, colorless
*V* = 495.76 (19) Å^3^	0.40 × 0.34 × 0.21 mm
*Z* = 2	

### 2-Amino-2-oxoethyl 4-nitrobenzoate (II) Data collection

**Table:**

Oxford Diffraction Xcalibur, Ruby diffractometer	1859 independent reflections
Radiation source: Enhance (Cu) X-ray Source	1560 reflections with *I* > 2σ(*I*)
Graphite monochromator	*R*_int_ = 0.018
Detector resolution: 10.2576 pixels mm^-1^	θ_max_ = 70.0°, θ_min_ = 4.7°
ω scans	*h* = −5→8
Absorption correction: multi-scan (SADABS; Bruker, 2008)	*k* = −8→8
*T*_min_ = 0.681, *T*_max_ = 0.797	*l* = −12→12
2971 measured reflections	

### 2-Amino-2-oxoethyl 4-nitrobenzoate (II) Refinement

**Table:**

Refinement on *F*^2^	Primary atom site location: structure-invariant direct methods
Least-squares matrix: full	Secondary atom site location: difference Fourier map
*R*[*F*^2^ > 2σ(*F*^2^)] = 0.050	Hydrogen site location: mixed
*wR*(*F*^2^) = 0.148	H atoms treated by a mixture of independent and constrained refinement
*S* = 1.06	*w* = 1/[σ^2^(*F*_o_^2^) + (0.080*P*)^2^ + 0.1088*P*] where *P* = (*F*_o_^2^ + 2*F*_c_^2^)/3
1859 reflections	(Δ/σ)_max_ < 0.001
153 parameters	Δρ_max_ = 0.24 e Å^−^^3^
0 restraints	Δρ_min_ = −0.29 e Å^−^^3^

### 2-Amino-2-oxoethyl 4-nitrobenzoate (II) Special details

**Table:**

Geometry. All esds (except the esd in the dihedral angle between two l.s. planes) are estimated using the full covariance matrix. The cell esds are taken into account individually in the estimation of esds in distances, angles and torsion angles; correlations between esds in cell parameters are only used when they are defined by crystal symmetry. An approximate (isotropic) treatment of cell esds is used for estimating esds involving l.s. planes.

### 2-Amino-2-oxoethyl 4-nitrobenzoate (II) Fractional atomic coordinates and isotropic or equivalent isotropic displacement parameters (Å^2^)

**Table:**

	*x*	*y*	*z*	*U*_iso_*/*U*_eq_	
O1	0.0727 (3)	−0.1039 (2)	0.29955 (18)	0.0820 (6)	
O2	0.19162 (19)	−0.00610 (18)	0.13179 (13)	0.0492 (4)	
O3	0.2532 (2)	−0.49315 (19)	−0.04910 (17)	0.0635 (5)	
O4	0.3147 (3)	0.8233 (2)	0.75383 (18)	0.0866 (6)	
O5	0.3833 (3)	0.9205 (2)	0.58393 (19)	0.0852 (6)	
N1	0.4638 (3)	−0.2474 (3)	0.0945 (2)	0.0608 (5)	
N2	0.3258 (2)	0.7948 (2)	0.62962 (19)	0.0526 (4)	
C1	0.1925 (2)	0.2274 (2)	0.35306 (18)	0.0409 (4)	
C2	0.2520 (3)	0.3754 (3)	0.30129 (19)	0.0443 (4)	
H2A	0.2634	0.3488	0.2061	0.053*	
C3	0.2945 (3)	0.5635 (3)	0.3920 (2)	0.0466 (4)	
H3A	0.3342	0.6645	0.3590	0.056*	
C4	0.2762 (2)	0.5962 (2)	0.53229 (19)	0.0425 (4)	
C5	0.2171 (3)	0.4526 (3)	0.58712 (19)	0.0465 (4)	
H5A	0.2065	0.4799	0.6825	0.056*	
C6	0.1739 (3)	0.2665 (3)	0.4952 (2)	0.0466 (4)	
H6A	0.1321	0.1666	0.5287	0.056*	
C7	0.1433 (3)	0.0225 (3)	0.2615 (2)	0.0465 (4)	
C8	0.1380 (3)	−0.1993 (3)	0.0349 (2)	0.0509 (5)	
H8A	0.0999	−0.1916	−0.0579	0.061*	
H8B	0.0285	−0.2604	0.0640	0.061*	
C9	0.2934 (3)	−0.3240 (2)	0.02512 (19)	0.0455 (4)	
H1	0.561 (3)	−0.323 (3)	0.082 (2)	0.057 (6)*	
H2	0.488 (3)	−0.127 (4)	0.138 (3)	0.062 (6)*	

### 2-Amino-2-oxoethyl 4-nitrobenzoate (II) Atomic displacement parameters (Å^2^)

**Table:**

	*U*^11^	*U*^22^	*U*^33^	*U*^12^	*U*^13^	*U*^23^
O1	0.1300 (16)	0.0426 (9)	0.0620 (10)	−0.0163 (9)	0.0267 (10)	0.0061 (7)
O2	0.0603 (8)	0.0354 (7)	0.0467 (7)	0.0097 (5)	0.0122 (6)	0.0032 (5)
O3	0.0579 (8)	0.0369 (7)	0.0759 (10)	0.0070 (6)	−0.0010 (7)	−0.0085 (7)
O4	0.1315 (16)	0.0536 (10)	0.0537 (10)	−0.0086 (10)	0.0189 (10)	−0.0075 (7)
O5	0.1262 (16)	0.0381 (8)	0.0797 (12)	−0.0066 (9)	0.0143 (11)	0.0079 (8)
N1	0.0543 (10)	0.0380 (9)	0.0710 (12)	0.0099 (8)	−0.0013 (8)	−0.0091 (8)
N2	0.0515 (9)	0.0380 (9)	0.0591 (10)	0.0061 (7)	0.0043 (7)	0.0030 (7)
C1	0.0403 (8)	0.0353 (9)	0.0443 (9)	0.0092 (7)	0.0064 (7)	0.0073 (7)
C2	0.0510 (10)	0.0394 (9)	0.0414 (9)	0.0097 (7)	0.0086 (7)	0.0094 (7)
C3	0.0522 (10)	0.0352 (9)	0.0528 (10)	0.0077 (7)	0.0077 (8)	0.0139 (8)
C4	0.0386 (8)	0.0343 (9)	0.0481 (10)	0.0078 (7)	0.0035 (7)	0.0032 (7)
C5	0.0504 (10)	0.0439 (10)	0.0406 (9)	0.0062 (7)	0.0086 (7)	0.0060 (7)
C6	0.0530 (10)	0.0385 (9)	0.0471 (10)	0.0045 (7)	0.0106 (8)	0.0116 (8)
C7	0.0527 (10)	0.0369 (10)	0.0464 (10)	0.0060 (7)	0.0078 (8)	0.0080 (8)
C8	0.0552 (11)	0.0402 (10)	0.0467 (10)	0.0088 (8)	0.0023 (8)	−0.0011 (8)
C9	0.0526 (10)	0.0347 (9)	0.0426 (9)	0.0053 (7)	0.0066 (7)	0.0027 (7)

### 2-Amino-2-oxoethyl 4-nitrobenzoate (II) Geometric parameters (Å, º)

**Table:**

O1—C7	1.190 (2)	C1—C7	1.492 (3)
O2—C7	1.339 (2)	C2—C3	1.390 (3)
O2—C8	1.445 (2)	C2—H2A	0.9300
O3—C9	1.228 (2)	C3—C4	1.378 (3)
O4—N2	1.213 (2)	C3—H3A	0.9300
O5—N2	1.203 (2)	C4—C5	1.379 (3)
N1—C9	1.320 (3)	C5—C6	1.383 (3)
N1—H1	0.93 (2)	C5—H5A	0.9300
N1—H2	0.85 (3)	C6—H6A	0.9300
N2—C4	1.474 (2)	C8—C9	1.506 (3)
C1—C2	1.387 (3)	C8—H8A	0.9700
C1—C6	1.391 (3)	C8—H8B	0.9700
			
C7—O2—C8	115.74 (15)	C4—C5—C6	117.62 (17)
C9—N1—H1	118.2 (14)	C4—C5—H5A	121.2
C9—N1—H2	120.2 (16)	C6—C5—H5A	121.2
H1—N1—H2	121 (2)	C5—C6—C1	120.57 (17)
O5—N2—O4	122.58 (18)	C5—C6—H6A	119.7
O5—N2—C4	118.83 (17)	C1—C6—H6A	119.7
O4—N2—C4	118.52 (17)	O1—C7—O2	123.05 (17)
C2—C1—C6	120.33 (17)	O1—C7—C1	124.14 (17)
C2—C1—C7	122.69 (16)	O2—C7—C1	112.80 (16)
C6—C1—C7	116.98 (16)	O2—C8—C9	114.12 (15)
C1—C2—C3	119.85 (17)	O2—C8—H8A	108.7
C1—C2—H2A	120.1	C9—C8—H8A	108.7
C3—C2—H2A	120.1	O2—C8—H8B	108.7
C4—C3—C2	118.12 (17)	C9—C8—H8B	108.7
C4—C3—H3A	120.9	H8A—C8—H8B	107.6
C2—C3—H3A	120.9	O3—C9—N1	123.64 (18)
C3—C4—C5	123.50 (16)	O3—C9—C8	117.23 (17)
C3—C4—N2	118.35 (17)	N1—C9—C8	119.14 (16)
C5—C4—N2	118.14 (17)		
			
C6—C1—C2—C3	0.4 (3)	C2—C1—C6—C5	−0.9 (3)
C7—C1—C2—C3	179.61 (16)	C7—C1—C6—C5	179.83 (16)
C1—C2—C3—C4	0.2 (3)	C8—O2—C7—O1	5.0 (3)
C2—C3—C4—C5	−0.4 (3)	C8—O2—C7—C1	−176.52 (14)
C2—C3—C4—N2	178.47 (15)	C2—C1—C7—O1	−171.5 (2)
O5—N2—C4—C3	−1.2 (3)	C6—C1—C7—O1	7.7 (3)
O4—N2—C4—C3	−178.06 (18)	C2—C1—C7—O2	10.0 (3)
O5—N2—C4—C5	177.72 (18)	C6—C1—C7—O2	−170.76 (15)
O4—N2—C4—C5	0.8 (3)	C7—O2—C8—C9	−95.53 (19)
C3—C4—C5—C6	−0.1 (3)	O2—C8—C9—O3	175.79 (17)
N2—C4—C5—C6	−178.96 (15)	O2—C8—C9—N1	−4.7 (3)
C4—C5—C6—C1	0.8 (3)		

### 2-Amino-2-oxoethyl 4-nitrobenzoate (II) Hydrogen-bond geometry (Å, º)

**Table:**

*D*—H···*A*	*D*—H	H···*A*	*D*···*A*	*D*—H···*A*
N1—H1···O3^i^	0.93 (2)	1.97 (2)	2.898 (2)	174 (2)

Symmetry code: (i) −*x*+1, −*y*−1, −*z*.

### 2-Amino-2-oxoethyl 4-aminobenzoate monohydrate (III) Crystal data

**Table:**

C_9_H_10_N_2_O_3_·H_2_O	*F*(000) = 448
*M**_r_* = 212.21	*D*_x_ = 1.329 Mg m^−^^3^
Monoclinic, *P*2_1_/*n*	Cu *K*α radiation, λ = 1.54184 Å
*a* = 8.2431 (16) Å	Cell parameters from 927 reflections
*b* = 4.8088 (10) Å	θ = 5.6–71.4°
*c* = 26.754 (5) Å	µ = 0.90 mm^−^^1^
β = 90.10 (3)°	*T* = 291 K
*V* = 1060.5 (4) Å^3^	Prismatic, colorless
*Z* = 4	0.28 × 0.24 × 0.17 mm

### 2-Amino-2-oxoethyl 4-aminobenzoate monohydrate (III) Data collection

**Table:**

Oxford Diffraction Xcalibur, Ruby diffractometer	2165 independent reflections
Radiation source: Enhance (Cu) X-ray Source	1129 reflections with *I* > 2σ(*I*)
Graphite monochromator	*R*_int_ = 0.055
Detector resolution: 10.2576 pixels mm^-1^	θ_max_ = 76.1°, θ_min_ = 3.3°
ω scans	*h* = −10→10
Absorption correction: multi-scan (SADABS; Bruker, 2008)	*k* = −4→5
*T*_min_ = 0.778, *T*_max_ = 0.859	*l* = −32→33
6444 measured reflections	

### 2-Amino-2-oxoethyl 4-aminobenzoate monohydrate (III) Refinement

**Table:**

Refinement on *F*^2^	Primary atom site location: structure-invariant direct methods
Least-squares matrix: full	Secondary atom site location: difference Fourier map
*R*[*F*^2^ > 2σ(*F*^2^)] = 0.053	Hydrogen site location: mixed
*wR*(*F*^2^) = 0.150	H atoms treated by a mixture of independent and constrained refinement
*S* = 0.99	*w* = 1/[σ^2^(*F*_o_^2^) + (0.055*P*)^2^] where *P* = (*F*_o_^2^ + 2*F*_c_^2^)/3
2165 reflections	(Δ/σ)_max_ < 0.001
160 parameters	Δρ_max_ = 0.16 e Å^−^^3^
0 restraints	Δρ_min_ = −0.24 e Å^−^^3^

### 2-Amino-2-oxoethyl 4-aminobenzoate monohydrate (III) Special details

**Table:**

Geometry. All esds (except the esd in the dihedral angle between two l.s. planes) are estimated using the full covariance matrix. The cell esds are taken into account individually in the estimation of esds in distances, angles and torsion angles; correlations between esds in cell parameters are only used when they are defined by crystal symmetry. An approximate (isotropic) treatment of cell esds is used for estimating esds involving l.s. planes.

### 2-Amino-2-oxoethyl 4-aminobenzoate monohydrate (III) Fractional atomic coordinates and isotropic or equivalent isotropic displacement parameters (Å^2^)

**Table:**

	*x*	*y*	*z*	*U*_iso_*/*U*_eq_	
O1	0.4903 (2)	0.0764 (5)	0.65718 (8)	0.0717 (6)	
O2	0.3607 (2)	0.3784 (4)	0.60830 (7)	0.0589 (5)	
O3	0.6302 (2)	0.8021 (4)	0.53839 (9)	0.0729 (6)	
N1	0.3578 (3)	0.7857 (6)	0.54142 (11)	0.0633 (7)	
N2	−0.2461 (3)	−0.2041 (6)	0.70993 (11)	0.0691 (8)	
C1	0.2029 (3)	0.0794 (5)	0.65846 (10)	0.0496 (6)	
C2	0.0592 (3)	0.1846 (6)	0.63861 (11)	0.0626 (8)	
H2A	0.0631	0.3213	0.6141	0.075*	
C3	−0.0884 (3)	0.0879 (6)	0.65503 (11)	0.0641 (8)	
H3A	−0.1833	0.1587	0.6411	0.077*	
C4	−0.0982 (3)	−0.1143 (6)	0.69216 (10)	0.0540 (7)	
C5	0.0452 (3)	−0.2176 (6)	0.71203 (11)	0.0592 (7)	
H5A	0.0413	−0.3527	0.7369	0.071*	
C6	0.1923 (3)	−0.1237 (6)	0.69558 (11)	0.0583 (7)	
H6A	0.2869	−0.1963	0.7094	0.070*	
C7	0.3628 (3)	0.1724 (6)	0.64262 (10)	0.0531 (7)	
C8	0.5179 (3)	0.4682 (6)	0.59167 (11)	0.0598 (7)	
H8A	0.5748	0.3129	0.5766	0.072*	
H8B	0.5809	0.5319	0.6201	0.072*	
C9	0.5026 (3)	0.6994 (6)	0.55425 (10)	0.0549 (7)	
O1W	0.9804 (4)	0.7452 (7)	0.52690 (16)	0.1083 (11)	
H1	0.348 (4)	0.917 (8)	0.5195 (15)	0.107 (14)*	
H2	0.264 (4)	0.697 (7)	0.5524 (12)	0.088 (11)*	
H3	−0.337 (4)	−0.147 (6)	0.6897 (12)	0.078 (10)*	
H4	−0.253 (4)	−0.365 (8)	0.7275 (15)	0.108 (14)*	
H1W	0.887 (5)	0.743 (8)	0.5326 (16)	0.100 (16)*	
H2W	0.939 (13)	0.731 (19)	0.495 (3)	0.35 (6)*	

### 2-Amino-2-oxoethyl 4-aminobenzoate monohydrate (III) Atomic displacement parameters (Å^2^)

**Table:**

	*U*^11^	*U*^22^	*U*^33^	*U*^12^	*U*^13^	*U*^23^
O1	0.0530 (12)	0.0881 (16)	0.0740 (14)	0.0039 (11)	0.0000 (10)	0.0249 (12)
O2	0.0543 (11)	0.0566 (11)	0.0659 (12)	0.0019 (9)	0.0084 (9)	0.0131 (9)
O3	0.0613 (13)	0.0704 (14)	0.0872 (15)	−0.0025 (11)	0.0146 (11)	0.0193 (12)
N1	0.0587 (16)	0.0643 (17)	0.0669 (17)	−0.0031 (13)	0.0020 (13)	0.0124 (13)
N2	0.0517 (15)	0.0773 (19)	0.0784 (19)	−0.0002 (14)	0.0039 (13)	0.0163 (15)
C1	0.0502 (15)	0.0501 (15)	0.0487 (14)	0.0018 (12)	0.0004 (11)	0.0015 (12)
C2	0.0592 (17)	0.0656 (19)	0.0632 (18)	0.0069 (14)	0.0038 (14)	0.0195 (15)
C3	0.0530 (17)	0.069 (2)	0.0699 (19)	0.0072 (14)	−0.0018 (14)	0.0149 (15)
C4	0.0531 (16)	0.0515 (15)	0.0572 (16)	0.0022 (13)	0.0046 (13)	0.0000 (13)
C5	0.0612 (18)	0.0587 (17)	0.0577 (17)	0.0028 (14)	0.0018 (14)	0.0136 (13)
C6	0.0532 (16)	0.0612 (17)	0.0603 (17)	0.0052 (14)	−0.0049 (13)	0.0077 (14)
C7	0.0569 (16)	0.0541 (16)	0.0483 (14)	0.0035 (13)	0.0044 (12)	−0.0002 (12)
C8	0.0555 (17)	0.0547 (17)	0.0694 (19)	0.0013 (13)	0.0076 (14)	0.0014 (14)
C9	0.0591 (17)	0.0485 (15)	0.0571 (16)	−0.0005 (13)	0.0087 (13)	−0.0012 (13)
O1W	0.0720 (19)	0.112 (2)	0.141 (3)	−0.0013 (17)	0.0223 (19)	−0.017 (2)

### 2-Amino-2-oxoethyl 4-aminobenzoate monohydrate (III) Geometric parameters (Å, º)

**Table:**

O1—C7	1.212 (3)	C2—C3	1.376 (4)
O2—C7	1.351 (3)	C2—H2A	0.9300
O2—C8	1.437 (3)	C3—C4	1.393 (4)
O3—O3	0.000 (7)	C3—H3A	0.9300
O3—C9	1.238 (3)	C4—C5	1.388 (4)
N1—C9	1.309 (4)	C5—C6	1.368 (4)
N1—H1	0.87 (4)	C5—H5A	0.9300
N1—H2	0.93 (4)	C6—H6A	0.9300
N2—C4	1.378 (4)	C8—C9	1.501 (4)
N2—H3	0.96 (3)	C8—H8A	0.9700
N2—H4	0.91 (4)	C8—H8B	0.9700
C1—C2	1.393 (4)	C9—O3	1.238 (3)
C1—C6	1.396 (4)	O1W—H1W	0.78 (4)
C1—C7	1.455 (4)	O1W—H2W	0.91 (9)
			
C7—O2—C8	114.9 (2)	C6—C5—H5A	119.5
C9—N1—H1	120 (2)	C4—C5—H5A	119.5
C9—N1—H2	122 (2)	C5—C6—C1	121.1 (3)
H1—N1—H2	118 (3)	C5—C6—H6A	119.4
C4—N2—H3	113.9 (19)	C1—C6—H6A	119.4
C4—N2—H4	120 (2)	O1—C7—O2	120.5 (3)
H3—N2—H4	119 (3)	O1—C7—C1	125.1 (3)
C2—C1—C6	118.1 (2)	O2—C7—C1	114.4 (2)
C2—C1—C7	123.2 (2)	O2—C8—C9	110.8 (2)
C6—C1—C7	118.7 (2)	O2—C8—H8A	109.5
C3—C2—C1	120.5 (3)	C9—C8—H8A	109.5
C3—C2—H2A	119.7	O2—C8—H8B	109.5
C1—C2—H2A	119.7	C9—C8—H8B	109.5
C2—C3—C4	121.1 (3)	H8A—C8—H8B	108.1
C2—C3—H3A	119.5	O3—C9—N1	124.0 (3)
C4—C3—H3A	119.5	O3—C9—N1	124.0 (3)
N2—C4—C5	120.6 (3)	O3—C9—C8	117.0 (3)
N2—C4—C3	121.1 (3)	O3—C9—C8	117.0 (3)
C5—C4—C3	118.2 (3)	N1—C9—C8	119.0 (3)
C6—C5—C4	120.9 (3)	H1W—O1W—H2W	79 (7)
			
C6—C1—C2—C3	−0.6 (5)	C8—O2—C7—O1	−0.1 (4)
C7—C1—C2—C3	179.9 (3)	C8—O2—C7—C1	−178.9 (2)
C1—C2—C3—C4	0.8 (5)	C2—C1—C7—O1	−176.2 (3)
C2—C3—C4—N2	177.6 (3)	C6—C1—C7—O1	4.3 (4)
C2—C3—C4—C5	−0.4 (5)	C2—C1—C7—O2	2.5 (4)
N2—C4—C5—C6	−178.1 (3)	C6—C1—C7—O2	−177.0 (2)
C3—C4—C5—C6	0.0 (4)	C7—O2—C8—C9	−179.2 (2)
C4—C5—C6—C1	0.2 (5)	O2—C8—C9—O3	177.4 (2)
C2—C1—C6—C5	0.1 (4)	O2—C8—C9—N1	−0.6 (4)
C7—C1—C6—C5	179.7 (3)		

### 2-Amino-2-oxoethyl 4-aminobenzoate monohydrate (III) Hydrogen-bond geometry (Å, º)

**Table:**

*D*—H···*A*	*D*—H	H···*A*	*D*···*A*	*D*—H···*A*
N1—H1···O3^i^	0.87 (4)	2.06 (4)	2.915 (3)	168
N2—H3···O1^ii^	0.96 (3)	1.98 (3)	2.919 (4)	163
O1*W*—H1*W*···O3	0.78 (4)	2.14 (4)	2.916 (4)	169 (4)
O1*W*—H2*W*···O1*W*^iii^	0.91 (9)	2.46 (9)	2.782 (7)	101

Symmetry codes: (i) −*x*+1, −*y*+2, −*z*+1; (ii) *x*−1, *y*, *z*; (iii) −*x*+2, −*y*+1, −*z*+1.

## References

[bb1] Allen, F. H., Kennard, O., Watson, D. G., Brammer, L., Orpen, A. G. & Taylor, R. (1987). *J. Chem. Soc. Perkin Trans. 2*, pp. S1–S19.

[bb2] Bruker (2008). *SADABS*. Bruker AXS Inc., Madison, Wisconsin, USA.

[bb3] Grell, J., Bernstein, J. & Tinhofer, G. (1999). *Acta Cryst.* B**55**, 1030–1043. 10.1107/s0108768199007120 10927445

[bb4] Groom, C. R., Bruno, I. J., Lightfoot, M. P. & Ward, S. C. (2016). *Acta Cryst.* B**72**, 171–179. 10.1107/S2052520616003954 PMC4822653 27048719

[bb5] Nishio, T., Nakajima, N., Kondo, M., Omote, Y. & Kaftory, M. (1984). *J. Chem. Soc. Perkin Trans. 1*, pp. 391–396.

[bb6] Parkin, A., Parsons, S., Robertson, J. H. & Tasker, P. A. (2002). *Acta Cryst.* E**58**, o1348–o1350.

[bb7] Rigaku OD (2018). *CrysAlis PRO*. Rigaku Oxford Diffraction, Yarnton, England.

[bb8] Saha, S. & Desiraju, G. R. (2017). *J. Am. Chem. Soc.* **139**, 1975–1983. 10.1021/jacs.6b11835 28080045

[bb9] Saha, S. & Desiraju, G. R. (2018). *Chem. Commun.* **54**, 6348–6351. 10.1039/c8cc02662a 29868675

[bb10] Santra, S. K., Banerjee, A., Rajamanickam, S., Khatun, N. & Patel, B. K. (2016). *Chem. Commun.* **52**, 4501–4504. 10.1039/c6cc00971a 26931492

[bb11] Sheldrick, G. M. (2008). *Acta Cryst.* A**64**, 112–122. 10.1107/S0108767307043930 18156677

[bb12] Sheldrick, G. M. (2015). *Acta Cryst.* C**71**, 3–8.

[bb13] Spek, A. L. (2020). *Acta Cryst.* E**76**, 1–11. 10.1107/S2056989019016244 PMC6944088 31921444

[bb14] Westrip, S. P. (2010). *J. Appl. Cryst.* **43**, 920–925.

[bb15] Wójcik, G., Mossakowska, I., Holband, J. & Bartkowiak, W. (2002). *Acta Cryst.* B**58**, 998–1004. 10.1107/s0108768102015033 12456978

